# Generation and characterisation of a *parkin-Pacrg* knockout mouse line and a *Pacrg* knockout mouse line

**DOI:** 10.1038/s41598-018-25766-1

**Published:** 2018-05-14

**Authors:** Sarah E. M. Stephenson, Timothy D. Aumann, Juliet M. Taylor, Jessica R. Riseley, Ruili Li, Jeffrey R. Mann, Doris Tomas, Paul J. Lockhart

**Affiliations:** 1 Bruce Lefroy Centre for Genetic Health Research, Murdoch Children’s Research Institute, Flemington Road, Parkville, Victoria Australia; 2 Department of Paediatrics, Faculty of Medicine, Dentistry and Health Sciences, The University of Melbourne, Parkville, Victoria Australia; 3 Florey Institute of Neuroscience and Mental Health, The University of Melbourne, Parkville, Victoria Australia; 4 Department of Pharmacology and Therapeutics, Faculty of Medicine, Dentistry and Health Sciences, The University of Melbourne, Parkville, Victoria, Australia; 5 Surgical Research, Murdoch Children’s Research Institute, Flemington Road, Parkville, Victoria Australia; 6 Monash Genome Modification Platform, Monash University, Clayton, Victoria Australia

## Abstract

Mutations in *PARK2* (*parkin*) can result in Parkinson’s disease (PD). *Parkin* shares a bidirectional promoter with *parkin coregulated gene* (*PACRG*) and the transcriptional start sites are separated by only ~200 bp. Bidirectionally regulated genes have been shown to function in common biological pathways. Mice lacking parkin have largely failed to recapitulate the dopaminergic neuronal loss and movement impairments seen in individuals with parkin-mediated PD. We aimed to investigate the function of PACRG and test the hypothesis that parkin and PACRG function in a common pathway by generating and characterizing two novel knockout mouse lines harbouring loss of both *parkin* and *Pacrg* or *Pacrg* alone. Successful modification of the targeted allele was confirmed at the genomic, transcriptional and steady state protein levels for both genes. At 18–20 months of age, there were no significant differences in the behaviour of parental and mutant lines when assessed by openfield, rotarod and balance beam. Subsequent neuropathological examination suggested there was no gross abnormality of the dopaminergic system in the substantia nigra and no significant difference in the number of dopaminergic neurons in either knockout model compared to wildtype mice.

## Introduction

Parkinson’s disease (PD) is an age associated progressive neurodegenerative movement disorder that is estimated to effect over 5 million people worldwide^1^. The movement symptoms of PD – bradykinesia, muscular rigidity, resting tremor or postural instability – result from the loss of dopaminergic neurons within the substantia nigra *pars compacta*. The mainstay of PD therapy for the last 60 years has remained dopamine replacement, however, no current clinical treatments are able to slow or halt the disease progression^2^.

For the most part, the aetiology of PD is unknown, and to date age is the only risk factor with significant evidence supporting a causal association with idiopathic PD^3^. An increased risk of PD has been identified in individuals who live in agricultural or rural areas, in particular farmers, which is thought to be related to pesticide exposure^4^. The causative associations of pesticides to PD aetiology are limited, perhaps owing to the heterogeneous nature of the disease, however long-term and low dose pesticide exposure is neurotoxic to dopaminergic neurons in a number of model systems^5^. For a small number (5–10%) of individuals who develop PD, the disorder can be directly attributed to mutation(s) in one of a handful of genes that have been demonstrated to cause PD^6,7^. The shared clinical and pathological features of idiopathic and gene-mediated PD is thought to be the result of a shared molecular aetiology, therefore considerable efforts has been directed to the development and characterisation of rodent and other models dysregulated for PD-associated genes^8^.

Mutation in *parkin* is the most common cause of early-onset autosomal recessive PD accounting for approximately 50% of all early-onset PD cases and between 15–20% percent of sporadic early-onset PD. In addition, mutations in *parkin* have also been linked to late onset sporadic disease^9–11^. *Parkin* encodes an E3 ubiquitin-protein ligases that functions in the covalent linkage of ubiquitin to specific substrates^12^. Parkin has been implicated in a number of cellular processes, including different pathways of protein and organelle degradation within the cell. Parkin was first shown to function in the ubiquitin proteasome system (UPS), which is the major cellular degradation pathway for short lived and misfolded proteins and involves the linkage of ubiquitin to specific lysine residues in the target proteins. This ubiquitin tag serves as a molecular signal recognised by the proteasome, resulting in the proteolysis of unwanted or damaged proteins^13^. Parkin mediates the formation of a lysine-48 polyubiquitin chain linked to the target protein, which functions as a signal for degradation by the proteasome. Parkin also is capable of alternative modes of ubiquitination including monoubiquitination and lysine-63 polyubiquitination. These modifications appear to function in signalling and autophagy, respectively^14,15^. More recently, Parkin and the PD-associated protein PTEN induced putative kinase 1 (PINK1) have been shown to play a key role in regulation of mitochondrial function via the autophagy of mitochondria, mitophagy^16^.

*Parkin* shares a bi-directional promoter with *parkin coregulated gene* (*PACRG*). The two genes are transcribed from a small (~200 bp) intergenic DNA region between the 5′ ends of genes that are arranged in an antisense orientation^17,18^. Bidirectional promoters are hypothesized to co-regulate expression of gene-pairs whose encoded proteins interact and/or function in the same biological pathway(s)^19–25^. PACRG does not possess any conserved protein domains that might provide evidence of protein function. However, a number of lines of evidence have suggested that PACRG plays a role in microtubule dynamics. Although evidence supporting a genetic association of *PACRG* mutation and PD has not been reported^26^, the protein has been shown to be a component of Lewy bodies in PD^27,28^ and steady state levels of parkin and PACRG have been shown to be inversely correlated with alpha-synuclein accumulation in the astrocytes of individuals with PD^29^. These observations suggest PACRG may be functionally coupled to parkin and involved in the cellular processes that characterizes neurodegeneration.

There is evidence linking the function of PACRG to parkin and autophagy. Parkin and PACRG have been shown to interact, and the interaction between the two proteins is potentiated by inhibition of the UPS^28^. Furthermore, steady state levels of PACRG are tightly regulated by the UPS^28,30^. PACRG interacts *in vitro* with tubulins and microtubules and PACRG orthologues localise to the centriole, a microtubule based structure at the core of the centrosome^31,32^. This structure nucleates and organises the microtubules of the cytoskeleton, which are necessary for aggresome formation^33^. Parkin has also been shown to interact with tubulins and microtubules and is directed to the centrosome via an interaction with histone deacetylase 6, which is a central component of basal autophagy that targets protein aggregates and damaged mitochondria^34–37^.

PACRG is a major component of aggresomes following proteasome inhibition, and over-expression of the protein significantly increased the number of aggresomes *in vitro*. In contrast, shRNA-mediated knockdown of *PACRG* decreased aggresome formation in response to proteasome inhibition, and the morphology of remaining aggresomes appeared diffuse compared to the typical cage-like morphology^27^. Moreover, PACRG, like parkin, plays a role in autophagy. Cell lines overexpressing PACRG display increased structural markers of autophagy such as autophagosomes as identified by transmission electron microscopy. By contrast, in cell lines with reduced steady state levels of PACRG, biochemical markers of autophagy including LC3-II and p62 are decreased. Furthermore, PACRG was also found to interact with p62, a protein involved in targeting aggresomes for autophagic removal^27^. These observations suggest that PACRG participates in the normal basal autophagic pathway with a direct correlation between steady state PACRG levels and activation of the autophagic pathway.

There is considerable *in vitro* data suggesting overlapping and linked functions for parkin and PACRG in microtubule dynamics and potentially the pathogenesis of PD. A number of *parkin* knockout models have been generated^38^, and the *quaking viable* spontaneous mutant is a naturally occurring knockout of *parkin* and *Pacrg* with *quaking* dysregulation^39–41^. However, *quaking viable* has a predominant dysmyelination phenotype caused by the dysregulation of *quaking* expression^42,43^ which complicates behavioural, neuropathological and biochemical analysis. Therefore, adequate mouse models to investigate the co-regulated role of parkin and PACRG, or of parkin in the absence of PACRG in these cellular processes are required to fully elucidate the function of these proteins.

Here we report the generation and characterisation of two novel knockout mouse line – a double *parkin-Pacrg* knockout and a single *Pacrg* knockout.

## Results and Discussion

### Generation of knockout mice

**Figure 1:**
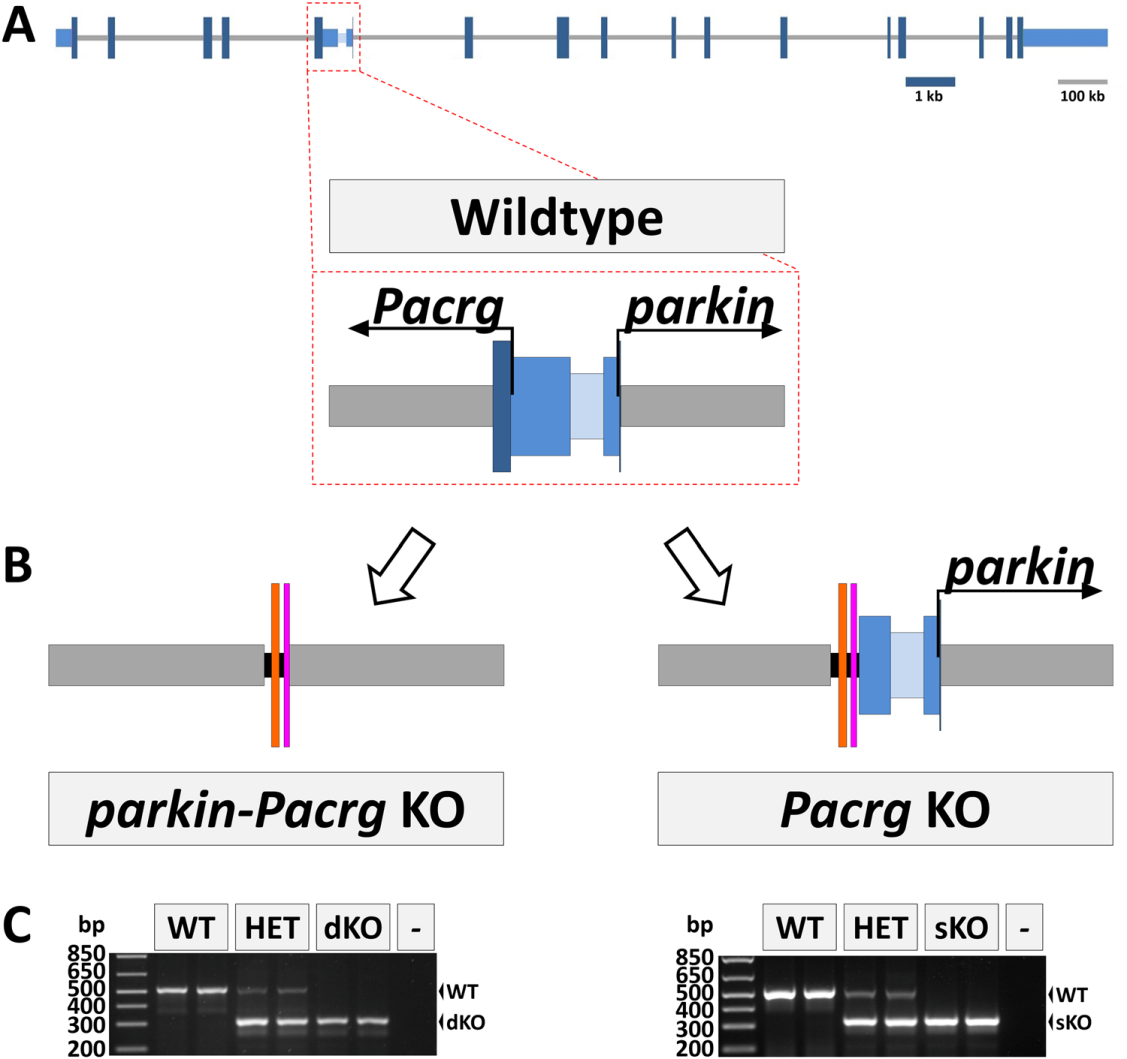
Generation of knockout mice. (**A**) Genomic structure of mouse *parkin* and *Pacrg* locus depicting the bidirectional genomic architecture of the genes. The dotted red box depicts a magnification of the bidirectional promoter and the first exons of both genes. (**B**) Schematic representation of knockout alleles. The double *parkin-Pacrg* knockout (dKO) lacks the entire bidirectional promoter and first exons of both genes. The single *Pacrg* knockout (sKO) lacks the entire coding sequence of the first exon of Pacrg and a portion of the 5′UTR sequence (from 121 bp upstream of *Pacrg*ATG to 211 bp downstream of *Pacrg*ATG (MGSCv37_chr17:11,032,521-11,032,855). (**C**) Generation of knockout mice was confirmed by PCR of genomic DNA (n = 2/genotype). Abbreviations: Wildtype (WT); Heterozygous (HET); double *parkin-Pacrg* knockout (dKO); single *Pacrg* knockout (sKO). Promoter DNA (light blue); 5′UTR DNA (mid blue); CDS (dark blue); FRT (orange); LoxP (pink); intron (gray); vector DNA (black).

*In vitro* studies suggest there are linked functions for parkin and PACRG in the pathogenesis of PD. Currently, existing mouse models are inadequate to investigate the co-regulated role of parkin and Pacrg to fully elucidate potential co-function of these proteins. Together murine *parkin* and *Pacrg* span almost 1.6 Mb of genomic sequence as a result of super expanded introns but their initiating methionine codons are separated by only 614 bp. Using traditional homologous recombination methods the entirety of the first coding exon of both *parkin* and *Pacrg* was deleted to generate the double *parkin-Pacrg* knockout (dKO). Likewise the single *Pacrg* knockout (sKO) was generated by deletion of the coding portion of the first *Pacrg* exon (Fig. 1 and Supplementary Figures 1–4).

**Figure 2:**
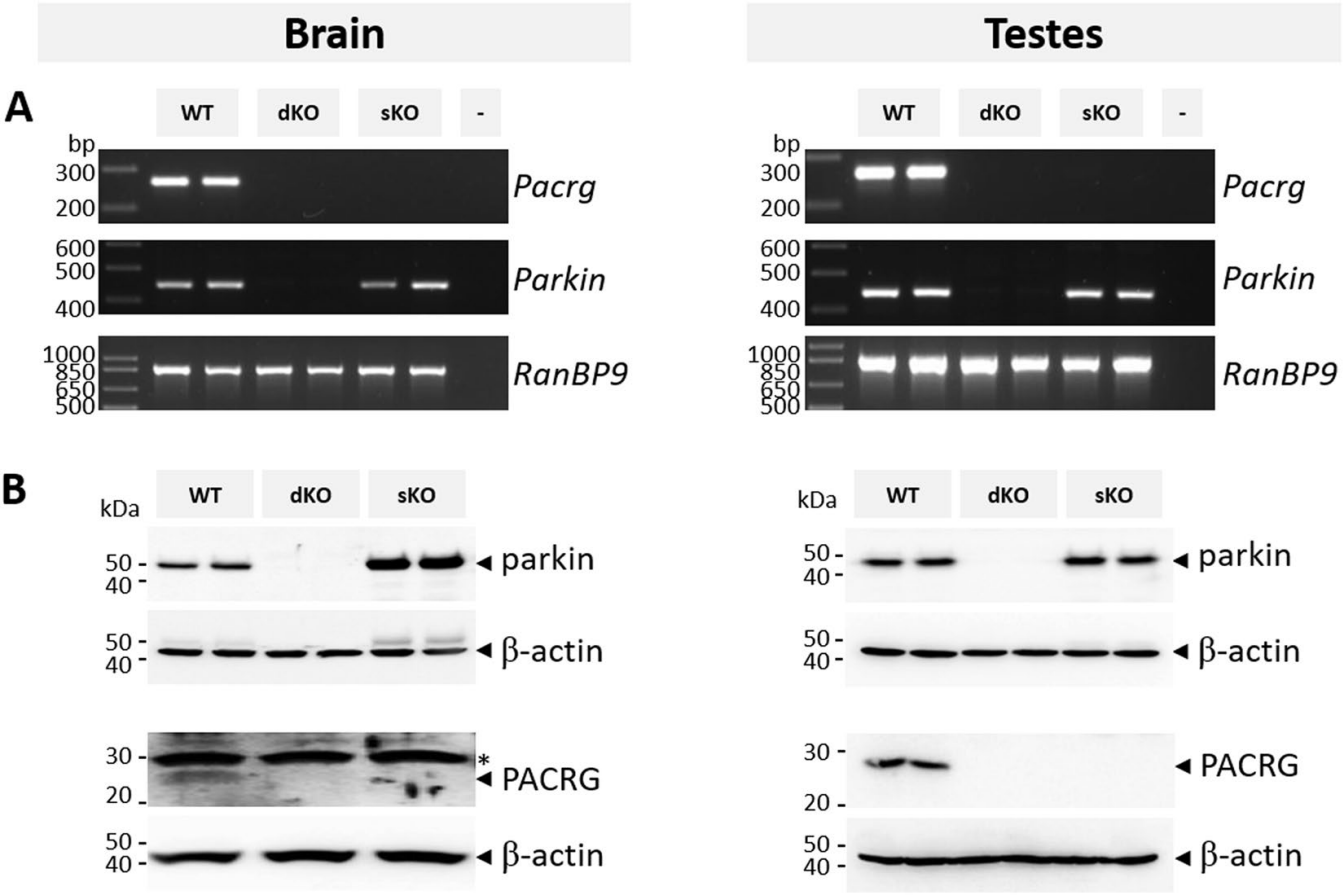
Knockout mice do not express protein from the deleted locus. (**A**) Total RNA from the brain and testes (n = 4/genotype) were assessed for expression of *parkin* and *Pacrg*. Expression of *parkin* and *pacrg* was not detected in the double *parkin-Pacrg* knockout (dKO), and expression of *Pacrg* was not detected in the single *Pacrg* knockout (sKO) but the full length transcripts were detected in wildtype (WT) tissues. Amplification of mRNA from the unrelated gene encoding *RAN binding protein 9* (*RanBP9*) was performed in parallel to confirm RNA integrity. (**B**) Whole protein lysates from the brain and testes (n ≥ 3/genotype) were used to investigate steady-state parkin and PACRG levels in WT, dKO and sKO mice. Western blot analysis using an anti-parkin antibody detected parkin in WT and sKO tissues but not in dKO tissues, while an anti-PACRG antibody detected PACRG only in the WT tissue. The membranes were reprobed with an anti-β-Actin antibody to confirm protein integrity and equivalent loading. *Non-specific band.

RT-PCR demonstrated that expression of both *parkin* and *Pacrg* was abrogated in the dKO, and that expression of *Pacrg* was abrogated in the sKO (Fig. 2A and Supplementary Figure 5). Western blot analysis confirmed that neither deletion allele encoded a full length or truncated protein (Fig. 2B and Supplementary Figure 6). Loss of *Pacrg* has previously been shown to cause hydrocephalus and infertility^44,45^. We identified enlargement of both the lateral (LV) and third ventricle (3 V) in the brains of both the sKO and dKO, and no male dKO or sKO mice were able to sire a litter. In addition, histological assessment indicated that elongated spermatids were largely absent from the lumen of both the dKO and sKO testes (Supplementary Figure 7). Collectively, these analyses confirmed the successful generation of two novel knockout mice lines; a double *parkin-Pacrg* knockout and a single *Pacrg* knockout.

### Steady state levels of parkin are elevated in the brain of sKO mice

**Figure 3:**
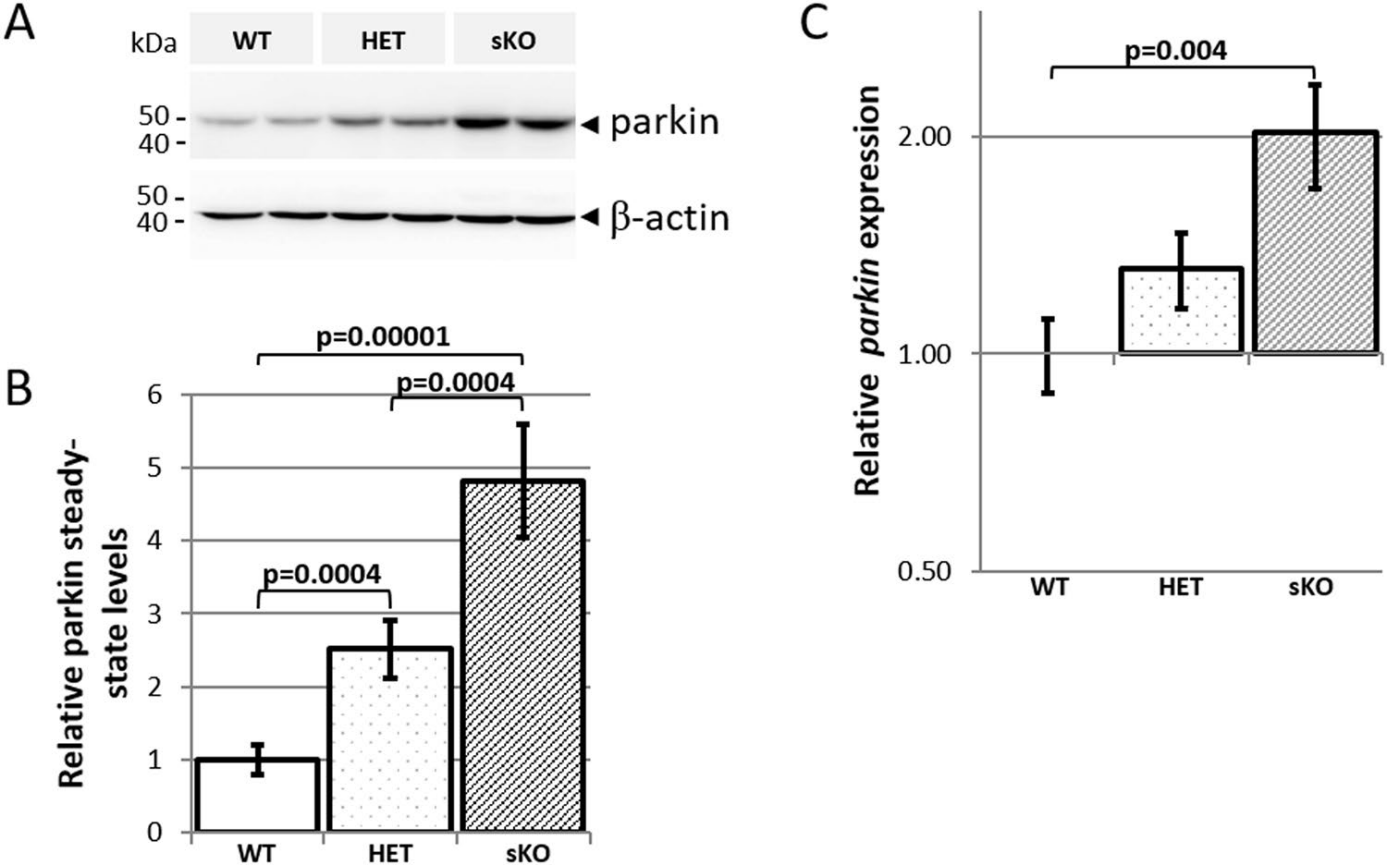
Single *Pacrg* knockout mice have increased parkin levels in the brain. Whole protein lysates from 8 week old mouse brains were used to determine the level of parkin in wildtype (WT), heterozygous (HET), and single *Pacrg* knockout (sKO) mice by western blot. (**A**) Representative western blot of parkin steady state levels in brain. The membranes were reprobed with an antibody directed against β-Actin to reference loading for digital quantitation of parkin steady state levels. (**B**) Mice (n ≥ 6/genotype) with the sKO allele have significantly increased parkin levels in the brain. (**C**) Quantitative RT-PCR was performed on RNA from 8 week old mouse brains to determine the level of *parkin* expression in WT, HET or sKO mice using exon spaning Taqman probes specific for *parkin* and *Gapdh* (control). Mice (n = 8/genotype) with two sKO alleles have significantly increased *parkin* expression. Error bars represent standard error of the mean. P values determined using the Student**’**s t-test (2 tailed, unequal variance).

Unexpectedly, the steady state level of parkin was elevated in sKO brain, but not the testes, compared to the wildtype brains (Fig. 2B). Using a larger cohort, which included all possible allele combinations we identified a 4.7 fold increase in the steady state level of parkin in the brain of sKO compared to wildtype (95% CI: 3.1–8.5, P = 0.00001, n ≥ 6/genotype) (Fig. 3A,B). There was no evidence for a sex difference in steady state levels of parkin (Supplementary Figure 8). Notably, the increase in the steady-state levels of parkin was also identified in the brains of mice heterozygous for the sKO allele compared to wildtype, 2.4 fold (CI: 1.6–4.3, P = 0.0004, n = 8/genotype). This demonstrates that the steady state level of parkin is significantly elevated in a copy number dependent manner.

We performed RT-qPCR to determine if elevated transcription of *parkin* in the sKO brain was the cause of the increase of steady state parkin (Fig. 3C). The expression of *parkin* was found to be 2.0 fold greater in the brain of mice homozygous for the sKO allele when compared to wildtype (95% CI: 1.2–3.1, P = 0.004, n = 8/genotype). The expression of *parkin* in the brain of mice heterozygous for the sKO allele when compared to wildtype was also elevated 1.3 fold but did not reach statistical significance (95% CI: 0.88–2.1, P = 0.1, n = 8/genotype). Collectively, this analysis suggests that increased expression of *parkin* contributes to the increase in steady state levels of parkin in the brain of sKO mice. We confirmed that elevated steady state parkin levels was not due to loss of Pacrg by reintroducing hemizygous transgenic expression of *Pacrg* into the sKO (Supplementary Figure 9).

There are several possible mechanisms that could result in the observed increase in *parkin* expression and steady state parkin in the single *Pacrg* knockout line. It is possible that the effect is predominantly due to loss of protein function, with loss of Pacrg directly affecting the production or turnover of parkin. However, there is no evidence to support this hypothesis. *In vitro* studies using cellular models have shown that parkin and PACRG interact, and the interaction is potentiated by proteasomal inhibition. Overexpression of parkin does not alter steady-state PACRG levels^28,30^ indicating that PACRG is not a substrate of the UPS-mediated ubiquitin ligase activity of parkin. Furthermore, over-expression of PACRG had no effect on steady-state parkin levels^28,30^. In addition, a direct effect of PACRG on parkin turnover does not account for the observation that expression of *parkin* mRNA is also significantly increased in the sKO mice (Fig. 3C). Therefore, the mechanism of elevated steady-state parkin in the sKO mouse generated here is likely to be altered transcription, rather than an effect of loss of PACRG on turnover of parkin. It is possible that the genomic modification associated with the targeting approach inadvertently upregulated *parkin* expression. However, the regions of the promoter suggested to be important for regulating expression of the highly conserved human *parkin* and *Pacrg* bidirectional promoter were unaltered by the targeting approach^17,18^. We believe the upregulated expression of *parkin* in the sKO is the result of the strains of animals used in the generation of the sKO strain. Specifically, the targeting construct and ES cells were derived from the 129S1/SvImJ mouse strain but the chimeric mice were subsequently mated to C57BL/6 J. Therefore, despite the ongoing backcross program, at least ~8 kb of sequence including the promoter/regulatory region of *parkin* is derived from the 129S1/SvImJ mouse strain in the sKO line. In contrast, the *parkin* allele of the wildtype mice is derived from the C57BL/6 J strain. Support for this conclusion is the published findings that demonstrated steady state parkin levels in the brain of 129 S were comparatively higher than C57BL/6^46^.

### Aged dKO or sKO mice do not show behavioural or neuropathological deficits of the dopaminergic system

PD typically develops later in life, is distinguished by a number of motor related symptoms, and is subsequently revealed to result from the loss of dopaminergic neurons within the substantia nigra *pars compacta* (SNpc) Therefore, aged mice (dKO (n = 9/genotype, 5 males, 18 months ± 4 weeks) and sKO (n = 10/genotype, 8 males, 19 months ± 8 weeks) and their age and sex-matched wildtype littermates) were subjected to a battery of behavioural tests and subsequent neuropathology assessment of the SNpc. The behavioural tests included open field to assess general locomotion and thigmotaxis; rotarod and walking beam to assess motor coordination and balance; grip strength to assess forelimb strength; footprint pattern analysis to identify deficiencies in gait; and buried food and faecal counts to identify prodromal symptoms. Consistent with previous studies of parkin knockout lines^38^, the results of these tests were largely unremarkable (Table 1 and Supplementary Figure 10). However, there was some evidence of dysfunction detected by gait analysis in both knockout strains. The stride length of both knockout strains was longer than the wildtype littermate, though it only reached statistical significance in the right forelimb of the double *parkin-Pacrg* knockout (P value = 0.04) and appeared slightly more pronounced in this strain. There was also some evidence that paw overlap may be affected in these strains, however, it only reached statistical significance on the right side of the single *Pacrg* knockout (P value = 0.03). Collectively, these slight deficiencies in gait locomotion may indicate some modest impairment of locomotion in these strains.

**Table 1:** Neuropathological and behavioural features of the knockout mice.

			Mean ± SEM			One-way ANOVA using Tukey’s multiple comparisons test								
			WT	dKO	sKO	WT vs dKO			WT vs sKO			dKO vs sKO		
			n = 19 (13 M)	n = 9 (5 M)	n = 10 (8 M)	Mean diff.	95% CI of diff.	Adjusted P Value	Mean diff.	95% CI of diff.	Adjusted P Value	Mean diff.	95% CI of diff.	Adjusted P Value
Age (m)			18.94 ± 0.40	18.2 ± 0.37	20.15 ± 0.64	0.67	−1.01 to 2.36	0.60	−1.21	−2.84 to 0.42	0.18	−1.89	−3.80 to 0.03	0.05
Weight (g)			40.61 ± 1.51	37.86 ± 1.48	38.16 ± 1.49	2.75	−2.91 to 8.41	0.47	2.46	−3.01 to 7.92	0.52	−0.29	−6.72 to 6.13	0.99
SN Stereology	TH + neurons		6211.58 ± 354.59	6265.11 ± 343.33	6736.70 ± 170.81	−53.53	−1284.88 to 1178.82	0.99	−525.10	−1713.96 to 663.72	0.53	−471.60	−1869.74 to 926.57	0.69
	Nissl + neurons		8619.95 ± 489.30	9253.89 ± 536.60	9852.1 ± 476.46	−633.94	−2490.71 to 1222.83	0.69	−1232.15	−3024.82 to 560.52	0.23	−598.21	−2706.51 to 1510.09	0.77
Open field	Distance travelled (m)		100.04 ± 6.27	95.83 ± 5.66	94.32 ± 6.02	3.04	−19.85 to 25.92	0.94	4.63	−17.46 to 26.71	0.87	1.59	−24.61 to 27.79	0.99
	Perimeter time (sec)		1607.20 ± 68.59	1686.72 ± 38.63	1493.79 ± 69.15	−79.46	−324.72 to 165.77	0.71	113.40	−123.35 to 350.18	0.48	192.90	−85.58 to 471.33	0.22
Rotarod	Latency to fall (sec)		123.58 ± 8.66	121.58 ± 9.54	139.52 ± 11.14	1.99	−32.87 to 36.85	0.99	−15.94	−49.60 to 17.72	0.49	−17.93	−57.52 to 21.65	0.52
Grip Strength	Peak tension (gf)		115.29 ± 5.76	125.56 ± 4.24	109.86 ± 7.73	−10.26	−32.72 to 12.20	0.51	5.44	−16.25 to 27.12	0.81	15.70	−9.81 to 41.20	0.30
Odour	Time to find food (sec)		117.58 ± 18.82	107.22 ± 13.63	130.5 ± 27.17	10.36	−64.68 to 85.39	0.94	−12.92	−85.37 to 59.53	0.90	−23.28	−108.48 to 61.92	0.78
Gut motility	Food (g/day)		10.66 ± 0.48	9.70 ± 1.30	10.38 ± 0.66	0.96	−1.62 to 3.54	0.64	0.34	−2.15 to 2.83	0.94	−0.62	−3.55 to 2.31	0.86
	Faecal pellets (no./day)		124.89 ± 4.38	113.44 ± 7.91	122.60 ± 3.82	11.45	−7.18 to 30.08	0.30	2.30	−15.69 to 20.28	0.95	−9.16	−30.31 to 12.00	0.55
Beam walking test	30 mm	Time to transverse (sec)	3.46 ± 0.25	3.71 ± 0.36	3.30 ± 0.21	−0.26	−1.24 to 0.72	0.80	0.16	−0.79 to 1.10	0.92	0.41	−0.70 to 1.52	0.64
		slips	0.63 ± 0.19	0.88 ± 0.41	0.20 ± 0.13	−0.26	−1.13 to 0.61	0.75	0.43	−0.41 to 1.27	0.43	0.69	−0.30 to 1.68	0.22
	18 mm	Time to transverse (sec)	3.40 ± 0.21	3.44 ± 0.28	3.18 ± 0.28	−0.04	−0.93 to 0.85	0.99	0.22	−0.64 to 1.08	0.80	0.26	−0.75 to 1.27	0.81
		slips	1.13 ± 0.23	1.39 ± 0.63	0.90 ± 0.35	−0.26	−1.53 to 1.01	0.87	0.23	−1.00 to 1.45	0.89	0.49	−1.00 to 1.93	0.69
	12 mm	Time to transverse (sec)	6.00 ± 0.60	5.34 ± 1.06	4.19 ± 0.49	0.66	−1.86 to 3.18	0.80	1.82	−0.61 to 4.25	0.17	1.16	−1.70 to 4.02	0.59
		slips	2.95 ± 0.55	3.11 ± 2.00	2.45 ± 1.25	−0.16	−4.02 to 3.70	0.99	0.50	−3.23 to 4.22	0.94	0.66	−3.72 to 5.04	0.93
Gait analysis	Forelimb stride length (mm)	Left	6.90 ± 0.21	7.56 ± 0.14	7.39 ± 0.19	−0.67	−1.41 to 0.07	0.08	−0.50	−1.21 to 0.21	0.21	0.17	−0.67 to 1.01	0.88
		Right	6.85 ± 0.21	7.59 ± 0.14	7.45 ± 0.15	−0.73	−1.44 to −0.02	0.04	−0.61	−1.30 to 0.07	0.09	0.12	−0.68 to 0.92	0.93
	Hindlimb stride length (cm)	Left	6.83 ± 0.20	7.50 ± 0.14	7.23 ± 0.23	−0.67	−1.43 to 0.08	0.09	−0.40	−1.13 to 0.32	0.37	0.27	−0.59 to 1.13	0.72
		Right	6.83 ± 0.21	7.52 ± 0.17	7.28 ± 0.19	−0.69	−1.45 to 0.07	0.08	−0.44	−1.17 to 0.30	0.32	0.25	−0.61 to 1.12	0.76
	Sway length (cm)	Forelimb	1.49 ± 0.04	1.43 ± 0.06	1.42 ± 0.05	0.08	−0.10 to 0.25	0.53	0.07	−0.10 to 0.24	0.58	−0.01	−0.21 to 0.19	1.00
		Hindlimb	3.03 ± 0.05	3.03 ± 0.09	2.86 ± 0.08	−0.01	−0.27 to 0.24	0.99	0.16	−0.08 to 0.41	0.25	0.17	−0.11 to 0.46	0.31
	Paw overlap (cm)	Left	1.35 ± 0.09	1.03 ± 0.12	1.05 ± 0.09	0.33	−0.03 to 0.67	0.07	0.31	−0.03 to 0.65	0.08	−0.02	−0.42 to 0.38	0.99
		Right	1.50 ± 0.10	1.34 ± 0.11	1.07 ± 0.11	0.14	−0.26 to 0.55	0.66	0.43	0.04 to 0.82	0.03	0.29	−0.17 to 0.74	0.29

Loss of dopaminergic neurons in the SNpc is a pathological hallmark of PD. To date, the majority of previously generated *parkin* knockout mice have not demonstrated significantly altered dopaminergic neuron populations compared to wildtype controls^38^. Stereological assessment of tyrosine hydroxylase positive neurons in the SNpc compared to wildtype, age and sex matched littermates did not identify any alterations in the morphology or number of dopaminergic neurons (Table 1 and Supplementary Figures 10–12).

Notably, we had difficulty acquiring female single *Pacrg* knockout mice for the study. A retrospective investigation of the sex and genotypes of litters suggested that the Mendelian ratios of knockout females was lower than expected (P value = 0.03, Supplementary Figure 13). Similar investigation of the double *parkin*-Pacrg knockout strain and the independent strain Quaking viable, which is a spontaneous mutant knockout of parkin and Pacrg and is also dysregulated for *quaking* expression, both demonstrated lower than expected numbers of knockout female (P = 0.003 and P = 0.042, respectively, Supplementary Figure 11). Therefore, harbouring two null *Pacrg* alleles appears to confer a female specific vulnerability to embryos/pre-wean pups. The mechanism underlying this phenomenon is not readily identifiable, but sexing and genotyping of day 1 pups to refine the window of susceptibility may be useful.

Bradykinesia, muscular rigidity, resting tremor or postural instability and loss of dopaminergic neurons in the substantia nigra *pars compacta* are the defining features of PD. Similar to other published *parkin* knockout mice, neither the double *parkin-Pacrg* knockout nor the single *Pacrg* knockout line display neuropathological or behavioural characteristics that recapitulate human disease. More broadly, the majority of PD mouse models have not proven robust at recapitulating the major pathological features of human PD^8^. However, collectively these studies suggest that gene mediated deficiency in the mouse may alter pathways common to human pathogenesis but for as yet unknown reasons do not cause a pronounced PD-like phenotype. Our new mouse models provide a platform to study the function of parkin and PACRG *in vivo*. Cellular models have delineated a role for parkin and PACRG in aggresome formation and resultant autophagic clearance. Therefore, these mice provide an opportunity to extend these studies and describe the consequence of loss of either parkin and/or Pacrg in an animal model.

## Materials and Methods

### Generation of knockout mice

All procedures using animals were conducted in accordance with the *Australian code of practice and use of animals for scientific purposes 7*^*th*^ *edition 2004* and approved by the Murdoch Children’s Research Institute Animal Ethics Committee. Mice were housed in temperature- and humidity-controlled rooms with an automatic 12/12 h light/dark cycle with food and water *ad libitum*. For all experiments, mice were directly compared to their wild-type littermates.

To generate a single *Pacrg* knockout mouse line and a double *parkin-Pacrg* knockout mouse line we used a homologous recombination approach that targeted the shared bidirectional promoter of *parkin* and *Pacrg* in 129S1/SvImJ embryonic stem cell (ES) line 2A-ES^47^. The targeting constructs were generated from 129S1/SvImJ genomic DNA. For the single *Pacrg* knockout (sKO) *LoxP* sites were incorporated within the 5′ untranslated regions (5′UTR) of the first exon and first intron of *Pacrg* to flank the encoded initiating methionine. For the double *parkin-Pacrg* knockout (dKO) *LoxP* sites were incorporated within the first intron of *Pacrg* and the first intron of *parkin* to flank the encoded initiating methionine of both *parkin* and *Pacrg* and the entire bidirectional promoter. To facilitate homologous recombination, homology arms of were generated from *parkin* intron one, 5′ targeting arm (~5.3 Kb) and from *Pacrg* intron one, 3′ targeting arm (~3.8 Kb). Positive and negative selection were incorporated to facilitate identification of correctly targeted clones. A neomycin phosphotransferase gene (*neo*) under the control of the thymidine kinase (TK) promoter and flanked by flippase recognition target (FRT) sites was incorporated downstream of the modified targeted allele and a diphtheria toxin gene (*DTA*) under the control of the Phosphoglycerate Kinase 1 gene (*PGK*) promoter was incorporated peripheral to the homology region (Supplementary Figure 1).

Targeted ES clones were generated following established protocols^48^. Following electroporation and selection, positively-targeted colonies were identified by Southern blot analysis (Supplementary Figure 2). Chromosome counts were performed to confirm euploidy (Supplementary Figure 3).

Congenic C57BL/6 J mice and outbred CD1® mice were purchased from the Walter and Eliza Hall Institute for Medical Research (WEHI) and blastocyst injection of the targeted 2A-ES cells was carried out in house. On the morning of blastocyst injection, the targeted ES cells (1 clone, ~1 × 106 cells) were thawed and resuspended in 10 ml of ES medium supplemented with 1 × 103 U/ml ESGRO® Mouse LIF Medium Supplement (Merck-Millipore, ESG1107). The cells were plated in the above culture onto a gelatinised 10 cm dish for 45 min, during which time the majority of STO-SNL2 feeder cells attached to the substrate and were removed. Floating ES cells were isolated by transferring the medium into a 15 ml Falcon tube and pelleted at 290 g for 5 min. Cells were resuspended in 1 ml blastocyst injection medium (ES medium without LIF and without β-mercaptoethanol, supplemented with 20 mM HEPES) and stored on ice until required.

Ten female C57BL/6 J mice at four weeks of age were prepared for superovulation by intraperitoneal injection of 5 IU Folligon® (Intervet), and 46 hours later 5 IU Chorulon® (Intervet). Each female was immediately mated with a male C57BL/6 J stud. Ovulation is expected approximately 12 hours post Chorulon®injection. Therefore, 14 hours after this injection, mice were culled by cervical dislocation and the oviducts removed and placed in 2 ml of M2 medium (Sigma-Aldrich, M7167) in a 3 cm Petri dish. Embryos at morulae stage were collected from oviducts and cultured overnight in EmbryoMax® KSOM Medium (Merck-Millipore, MR-106-D) in a 37 °C, 5% CO_2_ incubator. Blastocysts that developed from these cultured embryos were transferred to M2 medium overlaid with mineral oil on a glass slide mounted in the cold stage (14-16 °C) of a microinjection microscope. Approximately 8–12 2A-ES cells were injected into each blastocyst cavity. The blastocysts were then transferred into the oviduct of psuedopregnant CD1® female recipient mice at the equivalent of embryonic day (E) E2.5 (the result of mating CD1 females with vasectomised male mice, day of vaginal plug = E0.5).

Resulting chimeric pups were screened by PCR to identify potential founders using primers FRT-neoR (5′-CAGTTCATTCAGGGCACC-3′) and FRTneo F-Kpn1 (5′-ttttGGTACCGTGGATCCGCATGCGAAG-3′). Chimeric males were mated to C57BL/6 J females to confirm germline transmission and *neo* positive offspring were designated the founders of the lines.

Founder mice were mated to a C57BL/6 J mouse strain expressing both the Cre recombinase gene (Gt*(ROSA)26Sor-TgPGKCre)* and the FLP1 recombinase gene (Tg(ACTFLPe)9205Dym) to generate germline deletion of the *neoR* cassette and the loxP flanked targeted alleles. Successful deletion was confirmed by knockout allele and wildtype allele specific PCR. The single *Pacrg* knockout allele was differentiated from the wildtype allele using the primers mPACRG 5′ UTR F (5′-GCCTTTTAGAGTGTTTTCCC-3′) and mPACRG intron 1 R (5′-CCCTTACCAGTGAAACAGC-3′) which generated a 527 bp wildtype allele fragment or a 332 bp knockout allele fragment. The double *parkin*-*Pacrg* knockout allele was differentiated from the wildtype allele by PCR using a common reverse primer (mPACRG intron 1 R, 5′-CCCTTACCAGTGAAACAGC-3′) but different forward primers: mPACRG 5′UTR F (5′-GCCTTTTAGAGTGTTTTCCC-3′) was used to amplify a 527 bp wildtype allele fragment while KO 5′ arm F1 (5′-CAGGTGGCTCGGGTCGGC-3′) generated a 322 bp knockout allele fragment. Sequencing of the amplification product was used to confirm correct targeting (Supplementary Figure 4). A subsequent 6 generation breeding program was initiated with C57BL/6 J mice to remove the Cre and FLP transgenes and generate congenic lines. Transgenic mice over-expressing *Pacrg* under the control of the endogenous promoter have been described previously^49^.

### Characterisation of knockout mice

Mice were rendered unconscious with fluorinated anaesthetic and culled by cervical dislocation. Tissues for RNA/protein analysis were removed and immediately snap frozen in an Eppendorf tube on dry ice.

#### Expression analysis

RNA was extracted from frozen tissues using the RNeasy Mini Kit (74104, Qiagen) for end-point detection by RT-PCR or the SV Total RNA Isolation System (Z3105, Promega) for qPCR. The resultant RNA integrity was verified by electrophoresis in agarose, and the quantification and purity of nucleic acids determined using the NanoDrop 2000 UV-Vis Spectrophotometer (Thermo Fisher Scientific). To generate template for end-point detection by RT-PCR the Transcriptor First Strand cDNA Synthesis Kit (4897030001, Roche Applied Science) and the reactions were diluted 1:2 in water and 2 µl was used as template in PCR reactions. To generate template for qPCR analysis cDNA was generated using SuperScript® III First-Strand Synthesis System (18080051, Life Technologies) and diluted 2:5 in water and 2.5ul was used as a template in PCR reactions.

End-point detection by RT-PCR was preformed using performed using *Taq DNA Polymerase* and Q-Solution (201205, Qiagen) and a 57 °C to 52 °C touchdown protocol. A 264 bp fragment of *Pacrg* was amplified using primers spanning exon 1 (hPACRGE1FcDNA, 5′-GACAAGATGCCGAAGAGGAC-3′) and exon 2 (mPACRG-TgR, 5-CTTCTCAATCTCAACCTTCCAG-3′); and a 438 bp fragment of *parkin* was amplified using primers spanning exon 1 (mparkin-F, 5′-ATCGGCAGTTTGTCCACG-3′) and exon 2 (mparkin-F, 5′-ATCGGCAGTTTGTCCACG-3′). A 847 bp fragment of *Ranbp9* was amplified using primers spanning exon 8 (mRANBPM-F, 5′-CAGAAGTTGGTGTTAGCAGG-3′) and exon 14 (mRANBPM-R 5′- GCTTTGGCAGATTGTGGG-3′) to verify the integrity of the RNA/cDNA.

The expression of *parkin* was quantitated using TaqMan Assays spanning *parkin* exons 11–12 (Mm00450187_m1, Thermo Fisher Scientific) and the housekeeper assay mGapdh (4352339E, Thermo Fisher Scientific) in the FastStart TaqMan Probe Master (4673417001, Roche). The PCR program included a denaturation step of 10 min at 95 °C, followed by 45 cycles with a 15 sec denaturation at 95 °C and annealing at 60 °C for 1 min. Standard curves were generated from a dilution series using a calibrator sample, and contained six reference points to confirm linear amplification. All samples were assessed in triplicate in a single experiment. Relative expression was calculated using the comparative Ct method (∆∆Ct)^50^ and statistical significance was assessed by two-sample, two-tailed Student’s t-test and P < 0.05 was considered significant.

#### Western blot analysis

Frozen tissues were rapidly thawed and protein extracted by homogenisation in cold protein lysis buffer (10 mM Tris-HCl pH 7.4, 2% (w/v) SDS and 1× Complete Protease Inhibitor Cocktail (04693159001, Roche) by passing 10 times sequentially through 18 G, 21 G and 25 G needles. Each sample was then disrupted using the Digital Sonifier® Cell Disruptor 250 (Branson Ultrasonic Co.) with the following settings 0.02 s, amp 25%, pulse on 0.3 msec pulse off 0.7 msec. The soluble fraction was collected by centrifugation at 18,000 *g* for 30 min at 4 °C. Protein concentration was determined using a BCA Protein Assay Kit (23225, Thermo Fisher Scientific). Approximately 50 μg of lysate was fractioned in 15% SDS-PAGE gels that were subsequently transferred to PVDF Immobilon-P membranes (IPVH00010, Millipore-Merck). Membranes were incubated in blocking buffer (5% skim milk in TBS-Tween) for 1 hour at room temperature. Primary antibodies were incubate overnight at 4 °C. Parkin was detected using mouse anti-parkin ascites clone 8 (PKN8) diluted 1:1000^51^. PACRG was detected using rabbit anti-PACRG clone MC1290 diluted 1:1000^52^. β-Actin was used as a loading control and was detected by mouse anti-β-Actin ascites clone AC-15 diluted 1:10000 (A5441, Sigma-Aldrich). Antibody binding was revealed using peroxidase conjugated secondary antibodies donkey anti-rabbit (1:10,000 dilution, 711-035-152, Jackson) or donkey anti-mouse (1:10,000 dilution, 715-035-150, Jackson) with enhanced luminol-based chemiluminescent (ECL) Western Blotting Substrate (PIE32209, Thermo Scientific). Chemiluminescence was detected using the ImageQuant™ LAS 4000 Biomolecular imager (28-9558-10, GE Healthcare Life Sciences) with associated software. If the membrane required stripping before subsequent analysis with another primary antibody it was done so with Re-blot Plus Mild Antibody Stripping Solution (2502, Merck-Millipore). Images were analysed using ImageQuant TL 8.1 (29-0006-05, GE Healthcare Life Sciences). Densitometric intensity of detected bands was recorded for semiquantitative analysis between samples. Lanes and bands were identified automatically and then manually modified where appropriate; the rolling ball method was used to correct for background. Individual sample values were first determined by normalising the intensity of the protein of interest to the housekeeping control protein for each individual sample. To control for individual blot variation, each sample was then normalised to the same single sample included on each blot. The normalised value of each sample was then determined relative to wildtype, which was designated a value of 1. Interblot values were then combined and statistical significance was assessed by two-sample, two-tailed Student’s t-test and P < 0.05 was considered significant.

#### Structural analysis of the brain

Mice were culled by cervical dislocation and the brain removed and placed in 10% (v/v) Neutral Buffered Formalin. The samples were processed for paraffin embedding; Hematoxylin and eosin stain (H&E stain) histology and pathological assessment at the Australian Phenomics Network Histopathology and Organ Pathology Service, University of Melbourne.

#### Fertility and fecundity studies

To determine the fertility status of male mice, single *Pacrg* knockout mice or double *parkin-Pacrg* knockout mice males were individually co-caged with a fertile wildtype female mouse (C57BL/6 J) for at least six weeks. To analyse the male reproductive system and sperm production, mice were culled by cervical dislocation and the testes, epididymis and the vas deferens removed and placed in Bouin’s solution for ~20 hours at 4 °C. Samples were then placed in 10% Neutral Buffered Formalin and processed for paraffin embedding. Hematoxylin and eosin stain (H&E stain) histology and pathological assessment was performed at the Australian Phenomics Network Histopathology and Organ Pathology Service, University of Melbourne.

#### Behavioural Testing

Behavioural testing was conducted once using double *parkin-Pacrg* knockout mice their wild type age and sex matched littermates (n = 9/genotype, 5 males, 18 months ± 4 weeks) or single *Pacrg* knockout mice and their wild type age and sex matched littermates (n = 10/genotype, 8 males, 19 months ± 8 weeks). Behavioural analysis were conducted on both cohorts simultaneously, with each animal randomly assigned a number between 1 and 38, and tested in sequential order. Experimenters remained blinded to the genotypes of the animals during experimentation and data input.

Openfield: Locomotor activity was assessed in a standard 4-quadrant Open Field Box, 44 × 44 cm (Ugo-Basile). Mice were placed in individual chambers and behaviours were recorded via the video tracking software AnyMaze (Stoelting) over 60 minutes (5 minute time bins). The analysis included total distance travelled (cm) and distinguishing the time spent within an outer and inner zone of the open field (perimeter time).

Rotarod: Motor performance was assessed using the Rota Rod for Mice (47600, Ugo-Basile). The drum was slowly accelerated from a speed of 4 to 40 rpm over a 300 second duration. The latency to fall off the rotarod within this period was recorded by the Rota rod CUB software. One day before testing mice were habituated to the apparatus by giving them 5 unrecorded trials with a break of 30 min between each run. On the test day this was repeated and the latency to fall was recorded. The mean latency to fall off the rotarod was used for analysis.

Grip Strength: A grip strength meter (Columbus Instruments) was used to test mouse forearm grip strength as recorded in Newtons (N). Mice were held by the base of the tail and allowed to grip the trapeze with their front paws and then pulled with their body parallel to the bench. Each mouse was trailed 5 times and the readings averaged.

Buried food test: To stimulate hunger mice were fasted for 6 hours, with free access to water. After fasting the mice were introduced into a clean cage with bedding to acclimatise for 10 minutes. Subsequently, mice were transferred to their home cage while food was buried beneath the bedding in their new cage. Mice were then re-introduced into the new cage and the time taken to uncover and consume the buried food recorded.

Gut motility: Mice were housed individually over 72 hours. Food was weighed at the start of the experiment and every 24 hours, and faecal pellets were removed and counted every 24 hours. The average weight of food eaten per day and the average number of faecal pellets per day for each animal was used to determine gut motility.

Beam Walking Test: Fine motor coordination was tested using the beam walking test as described previously^53^. Mice were placed at one end of an elevated narrow beam where they had to remain upright and to walk 80 cm to the safety of a secluded platform. The beam was located 50 cm above a padded floor. Animals were successively tested on 3 different square edged beams with a diameter of 30, 18, and 12 mm. Mice were tested in two consecutive trials on each beam. Mice were placed on the beam ~100 cm from the secluded platform and the time taken to transverse 80 cm of the beam, as predefined on the beam, was recorded. A camera captured the animal from behind as it traversed the beam and the number of footslips was determined. For each measurement, the mean scores of the two trials for each beam were used in the analysis.

Gait analysis: The hindpaws were coated in blue paint and the forepaws were coated in pink paint, and the animal was allowed to walk along a narrow (5 cm wide, 50 cm long, 10 cm high) paper-covered corridor leaving a track of footprints. Animals were given 5 trails with a break of 1 hour between each run^53^. The footprint patterns were scanned and distances between footprints measured using Kinovea Software v8.25 (www.kinovea.org). Four measurements were ascertained: stride length, hindbase width, frontbase width, and front/hind footprint overlap^54^.

Statistics: For both behavioural and neuropathological analysis (outlined below), comparison of the three groups with one-way ANOVA using Tukey’s Multiple Comparison test where P < 0.05 was considered significant were performed using the software GraphPad Prism version 7.03.

#### Tyrosine hydroxylase immunohistochemistry

All animals used in behavioural experiments were included for stereological investigation. Animals were culled with sodium pentobarbitone (100 mg/kg i.p.) and intracardially perfused with 37 °C heparinized phosphate-buffered saline (PBS) followed by 4% paraformaldehyde in PBS (1.15 mL/g body weight). The brain was removed and placed in PBS containing 30% sucrose for 2–3 days. Coronal cryostat sections (16 μm) were cut through the midbrain and mounted on gelatinized microscope slides. Every fifth section was incubated in 5% normal goat serum and 0.3% triton X-100 in PBS (30 min), immunoreacted with polyclonal rabbit anti-TH (1:400, Chemicon, Temecula, CA, USA, 48 h), polyclonal biotinylated goat anti-rabbit (1:1000, Dako, Carpinteria, CA, USA, 2 h), avidin-peroxidase (1:500, 1 h), cobalt and nickel intensified diamino-benzidine (0.5 mg/mL, 15 min), then hydrogen peroxide (0.01%, 3–5 min). Sections were Nissl stained (neutral red), dehydrated in alcohol, cleared (X-3B) and coverslipped.

#### Stereology

Stereology was performed using a Leica DMLB stereology microscope (Stereo Investigator, Micro-BrightField, Williston, VT, USA). Using a 4× objective lens and 10× ocular lenses the boundaries of substantia nigra *pars compacta* (SNpc) were identified and delineated via anatomical landmarks and TH+ cell density differences between SNpc and adjacent midbrain catecholaminergic nuclei (ventral tegmental area and retrorubral field). The number of TH+ neurons and total neurons (Nissl+ cells excluding those with soma diameter <5 μm, which were assumed to be glia) in the SNpc were estimated using unbiased stereological methods. Specifically, using a 40×/1.00na oil objective lens and 10× ocular lenses counts of TH+ neurons and total neurons within a counting frame (45 × 35 μm = 1575 μm^2^) were made at regular pre-determined intervals (x = 140 μm, y = 140 μm) throughout the SNpc in every fifth section. Only neurons with a visible nucleus were counted. SNpc neurons in different brains were counted by the same person who was blind to genotype over successive days until all stereology was complete^55^.

#### Data availability

All data generated or analysed during this study are included in this published article (and its Supplementary Information files).

## Electronic supplementary material

Dataset 1
