# Supplementary material for: Generation and characterisation of a parkin-Pacrg knockout mouse line and a Pacrg knockout mouse line
Source: Sci Rep. 2018 May 14;8:7528. doi: 10.1038/s41598-018-25766-1 (PMC5951884; doi:10.1038/s41598-018-25766-1)
Supplement: Supplementary file 1 — Dataset 1 [file 41598_2018_25766_MOESM1_ESM.docx]

Generation and characterisation of a *parkin-PACRG* knockout mouse line and a *PACRG* knockout mouse line.

Sarah E.M. Stephenson^1,2^, Timothy D. Aumann^3^, Juliet M. Taylor^4^, Jessica R. Riseley^1^, Ruili Li^5^, Jeffrey R. Mann^6^, Doris Tomas^3^, Paul J. Lockhart^1,2*^.

^1^Bruce Lefroy Centre for Genetic Health Research, Murdoch Children’s Research Institute, Flemington Road, Parkville, Victoria, Australia.

^2^Department of Paediatrics, Faculty of Medicine, Dentistry and Health Sciences, The University of Melbourne, Parkville, Victoria, Australia.

^3^Florey Institute of Neuroscience and Mental Health, The University of Melbourne, Parkville, Victoria, Australia.

^4^Department of Pharmacology and Therapeutics, Faculty of Medicine, Dentistry and Health Sciences, The University of Melbourne, Parkville, Victoria, Australia.

^5^Surgical Research, Murdoch Children’s Research Institute, Flemington Road, Parkville, Victoria, Australia.

^6^Monash Genome Modification Platform, Monash University, Clayton, Victoria, Australia.

^*^Corresponding author

Email: [paul.lockhart@mcri.edu.au](mailto:paul.lockhart@mcri.edu.au)


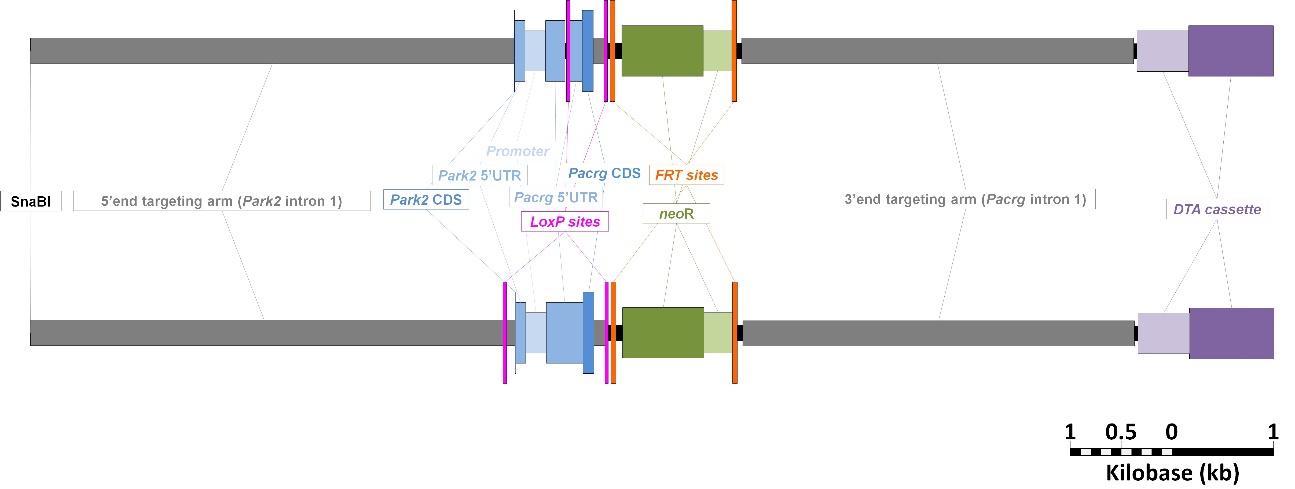


Supplementary Figure 1. Knockout constructs used in targeting experiments. Targeting constructs were linearized via an SnaBI site (5’ end) and a Diphtheria A toxin (DTA) encoding cassette (3’ end) was used increase efficiency. Top: Single *Pacrg* knockout targeting construct, bottom: double *parkin-Pacrg* knockout targeting construct.

Supplementary Figure 2. Identification of correctly targeted ES clones. Schematic representation of the wild type, *parkin*-*Pacrg* knockout and *Pacrg* knockout alleles. Details of the probe locations, restriction enzyme cleavage sites and predicted fragments sizes are indicated. Southern blot analysis of targeted 2A-ES cells. A) 5’ probe, B) 3’ probe and, C) neo probe to test for a single genomic insertion. For the single *Pacrg* knockout the targeted allele generates fragments of 10.279 kb, 9.687 kb and 1.453 kb, respectively. For the double *parkin-Pacrg* knockout the targeted allele generates fragments of 10.264 kb, 9.687 kb and 6.744 kb, respectively.


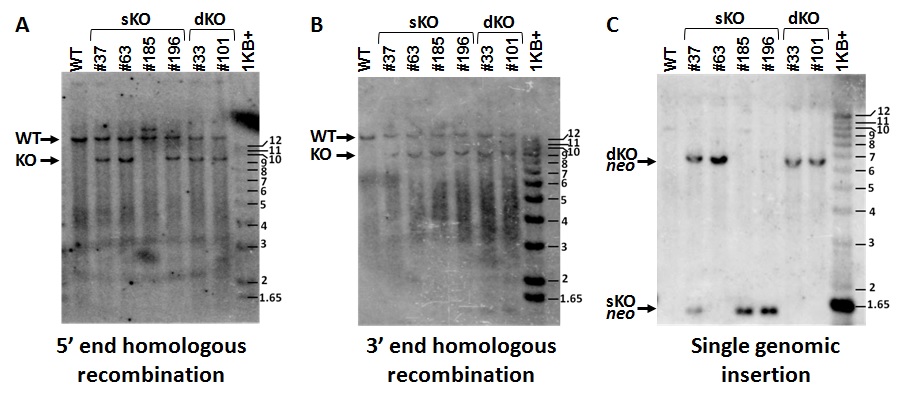

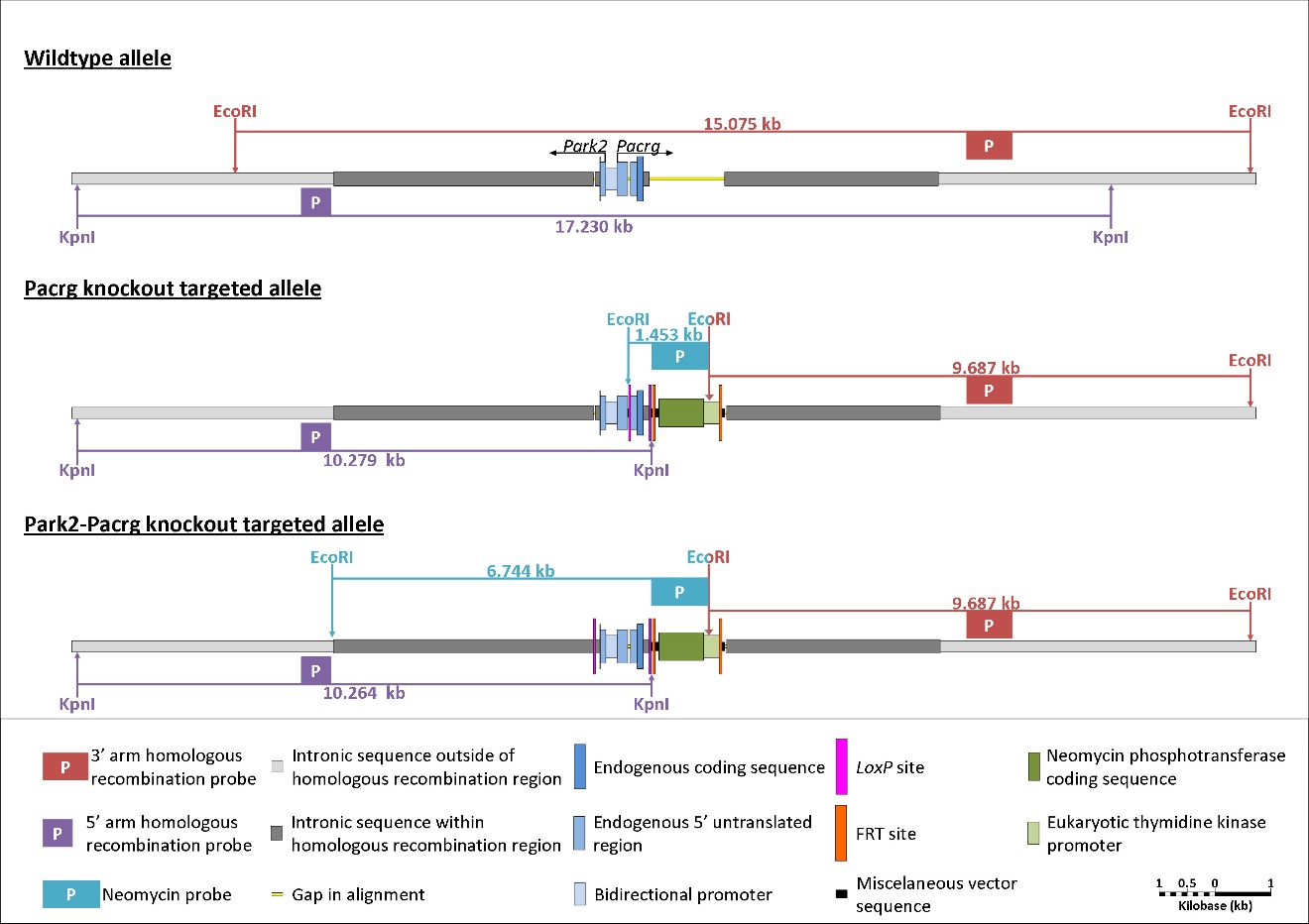


Supplementary Figure 3. Analysis of euploid chromosome number in the targeted ES clones. A) Representative image of a metaphase spread generated from the targeted ES cells. The number of times each chromosome number was identified in a metaphase spread of the single *Pacrg* knockout targeted ES clone (B) or the double *parkin-Pacrg* knockout targeted ES clone (C) is shown. Greater than 100 independent spreads were counted per clone.


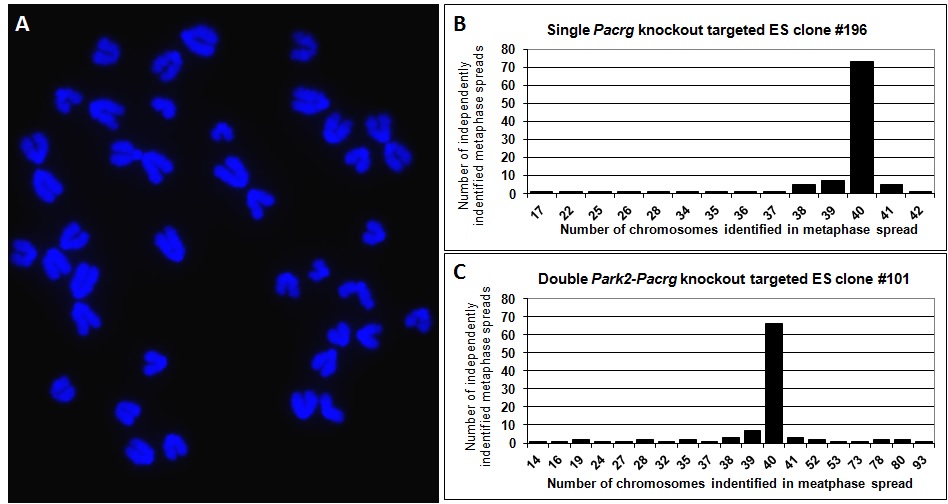

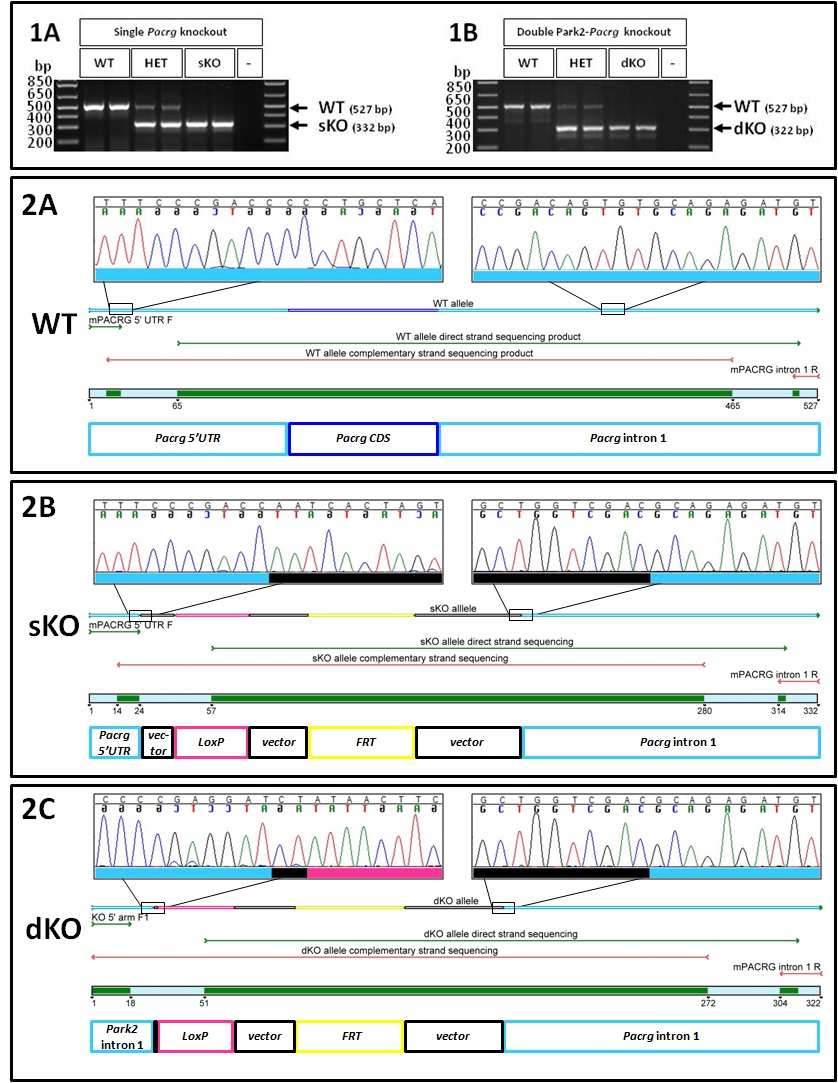


**Supplementary Figure 4. Genotyping of knockout mice.** 1) PCR protocol used to differentiate knockout allele(s) from wildtype allele. 2) Sequencing of wildtype (WT), single *Pacrg* knockout (sKO) and double *parkin*-*Pacrg* knockout (dKO) alleles confirmed that PCR products encoded the correct sequence (Alignments shown were generated with Sequencher analysis software).

**Supplementary Figure 5.** **Knockout mice do not express the deleted allele**. Total RNA from the brain and testes were assessed for expression of *parkin* and *Pacrg*. Expression of *parkin* and *pacrg* was not detected in the double *parkin-Pacrg* knockout (dKO), and expression of *Pacrg* was not detected in the single *Pacrg* knockout (sKO) but the full length transcript was detected in wildtype (WT). Amplification of mRNA from the unrelated gene encoding RAN binding protein 9 (*RanBP9*) was performed in parallel to confirm RNA integrity.


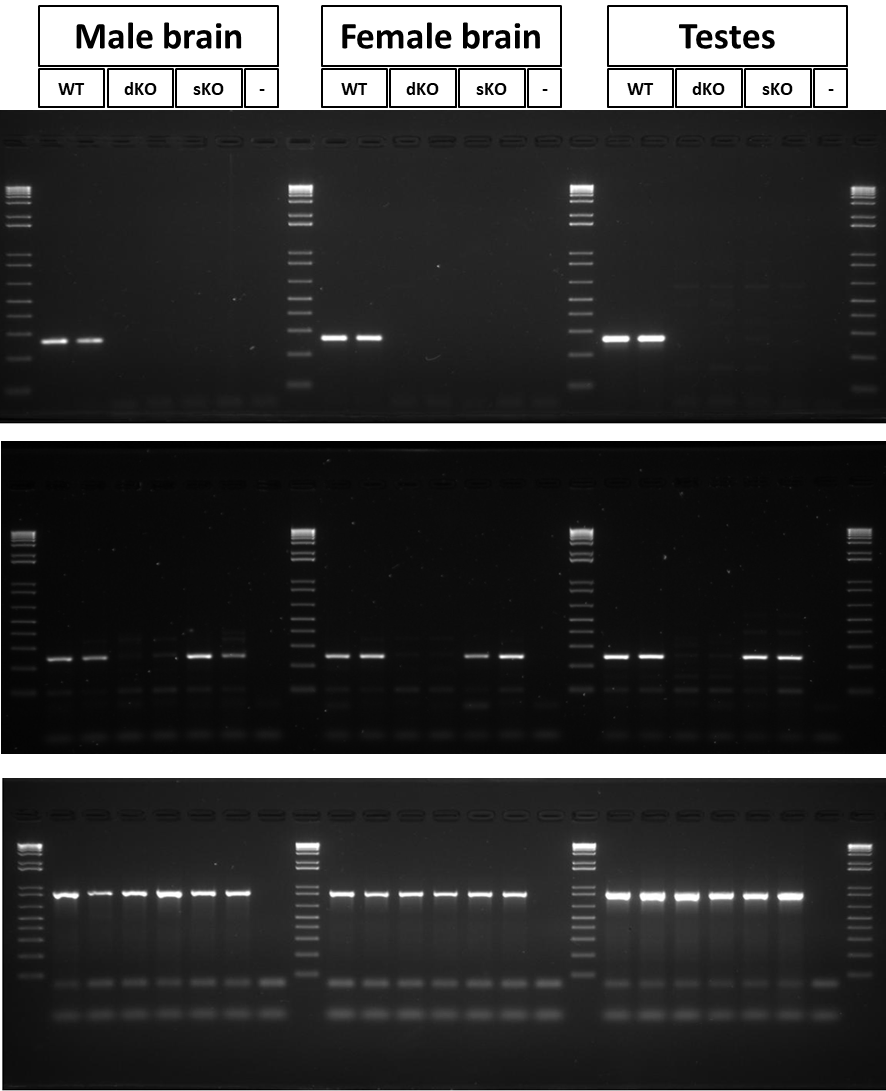


*parkin*

*Pacrg*

*Ranbpm*

**Supplementary Figure 6.** **Knockout mice do not express protein from the deleted locus**. Whole protein lysates from the brain and testes were used to investigate steady-state parkin and Pacrg levels in wildtype (WT), double *parkin-Pacrg* knockout (dKO) and single *Pacrg* Knockout (sKO). Western blot analysis using an anti-parkin antibody detected parkin in WT and sKO tissues but not in dKO tissues, while an anti-PACRG antibody detected PACRG only in the WT tissue. The membranes were reprobed with an anti-β-Actin antibody to confirm protein integrity and equivalent loading.


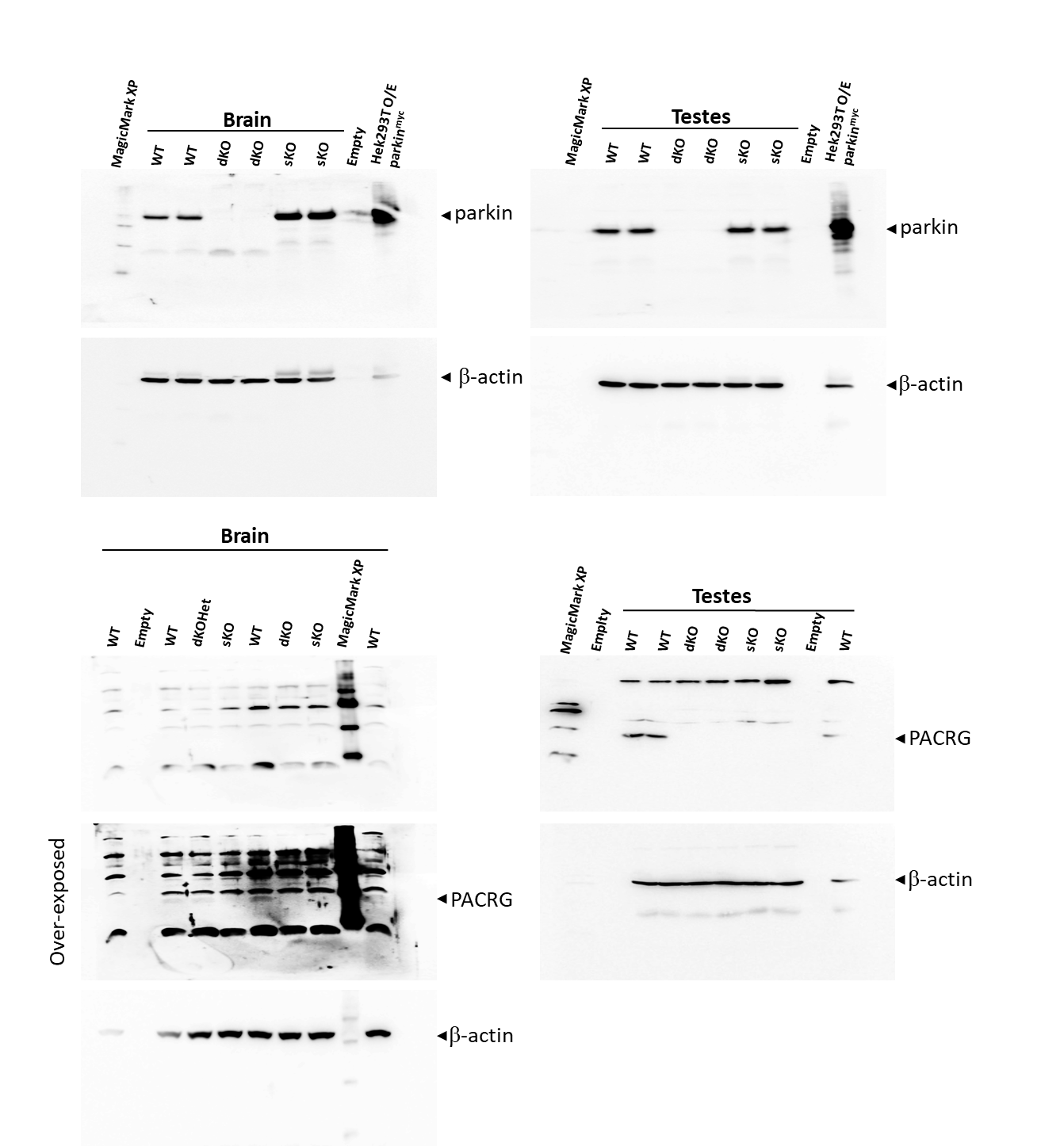


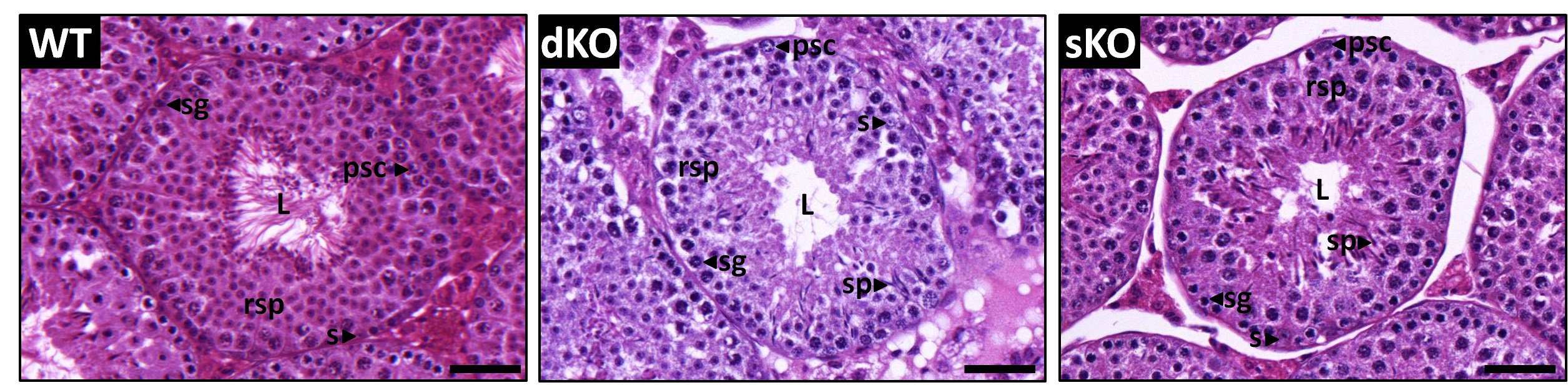


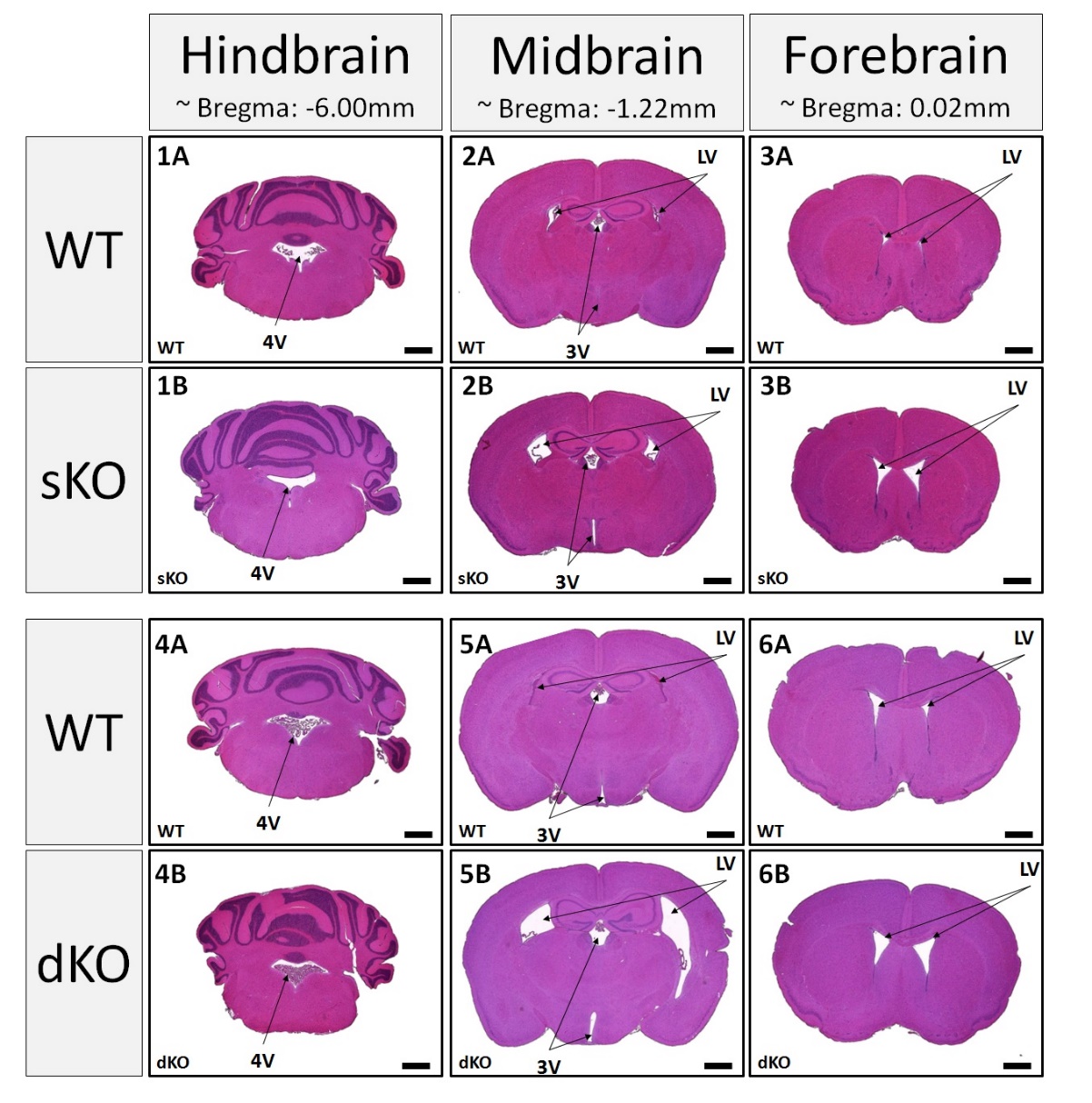


**Supplementary Figure 7. Knockout mice have enlarged lateral ventricles and lack enlongated spermatids.** A) Hematoxylin and eosin stained coronal brain sections prepared from three levels of the mouse brain (n=3/genotype). Hindbrain: includes the cerebellum, pons and fourth ventricle (4V), approximate Bregma: -6.00 mm. Midbrain: includes the thalamus, hypothalamus, lateral ventricles (LV) and third ventricle (3V), approximate Bregma: -1.22 mm. Forebrain: including cingulate cortex, frontal cortex, parietal cortex, piriform cortex, corpus callosum, LV, caudate putamen, ventral median fissure and anterior commissure, approximate Bregma: 0.02 mm. Each section from the mutant mice is shown with an age and sex matched section from a wildtype animal. Scale bar 1 mm. B) Hematoxylin and eosin stain was used to characterise testes tissue (n= 2/genotype). The testes of both knockout mice showed a reduced number of mature elongated spermatids in the lumen (L). In both knockout mice, the majority of identified spermatids had morphological features consistent with features of stages 11-13 of spermatid (sp) development. Spermatogonia (sg), Sertoli cells (s), pachytene spermatocytes (psc) and round spermacytes (rsc) were identifiable indicating early spermatogenesis had occurred. Scale bar 50μm. Abbreviations: Single *Pacrg* knockout (sKO); double *parkin-Pacrg* knockout (dKO); wildtype (WT).

**A**

**B**

Supplementary Figure 8. Increased parkin levels in the brain are not affected by gender. Whole protein lysates from 8 week old mouse brains were used to determine the level of parkin in wildtype (WT/WT), single *Pacrg* knockout (sKO/sKO) and heterozygous (WT/sKO) mice by western blot. The membranes were reprobed with β-Actin to reference loading for digital quantitation of parkin steady state levels. The elevated level of parkin is not affected by gender (male, blue; female, pink). Error bars represent standard error of the mean.


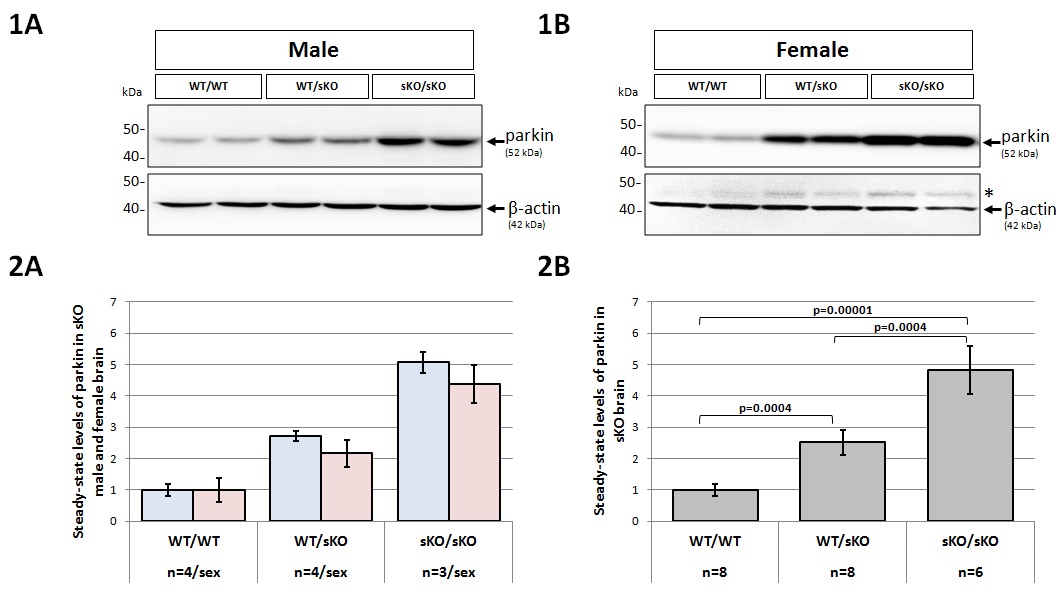

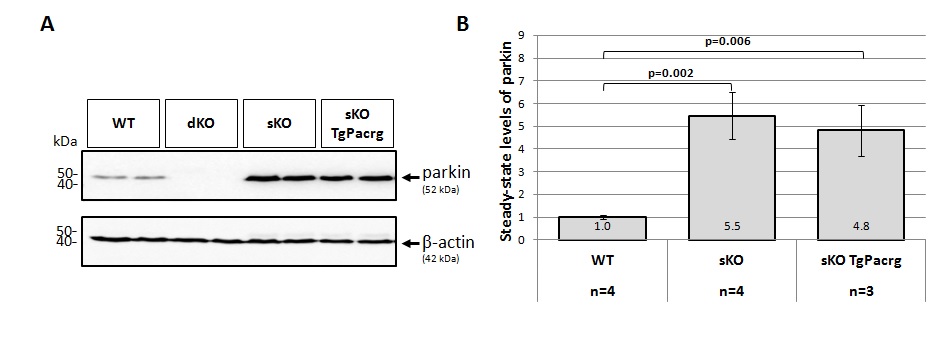


Supplementary Figure 9. Increased parkin levels in the brain of single *Pacrg* knockout are not altered by transgenic expression of PACRG. Whole protein lysates from 8 week old mouse brains were analysed to determine the steady state level of parkin in wildtype (WT), double *parkin-Pacrg* knockout (dKO), single *Pacrg* knockout (sKO) and single *Pacrg* knockout mice with hemizygous transgenic expression of *Pacrg* (sKO-TgPacrg) A) Representative Western blot of parkin steady state levels. The membranes were reprobed (without stripping) with β-Actin to reference as a loading control for quantitation. B) Ratio of parkin:β-actin determined by quantitation of Western blots. Error bars represent standard error of the mean. P values determined using the Student’s t-test (2 tailed, unequal variance).

**Supplementary Figure 10.** **Neuropathological and behavioural features of knockout mice.** Aged mice (dKO (n= 9/genotype, 5 males, 18 months ± 4 weeks) and sKO (n=10/genotype, 8 males, 19 months ± 8 weeks)) were subjected to a battery of tests to determine whether any behavioural motor deficits existed between knockout mice and their age and sex-matched wildtype littermates. These tests included open field to assess general locomotion and thigmotaxis; rotarod and walking beam to assess motor coordination and balance; grip strength to assess forelimb strength; footprint pattern analysis to identify deficiencies in gait; and buried food and faecal counts to identify prodromal symptoms. The outcome of comparison of the three groups with one-way ANOVA using Tukey’s Multiple Comparison test is shown in Table I. Male mice are indicated by blue dots and female mice are indicated by red dots.


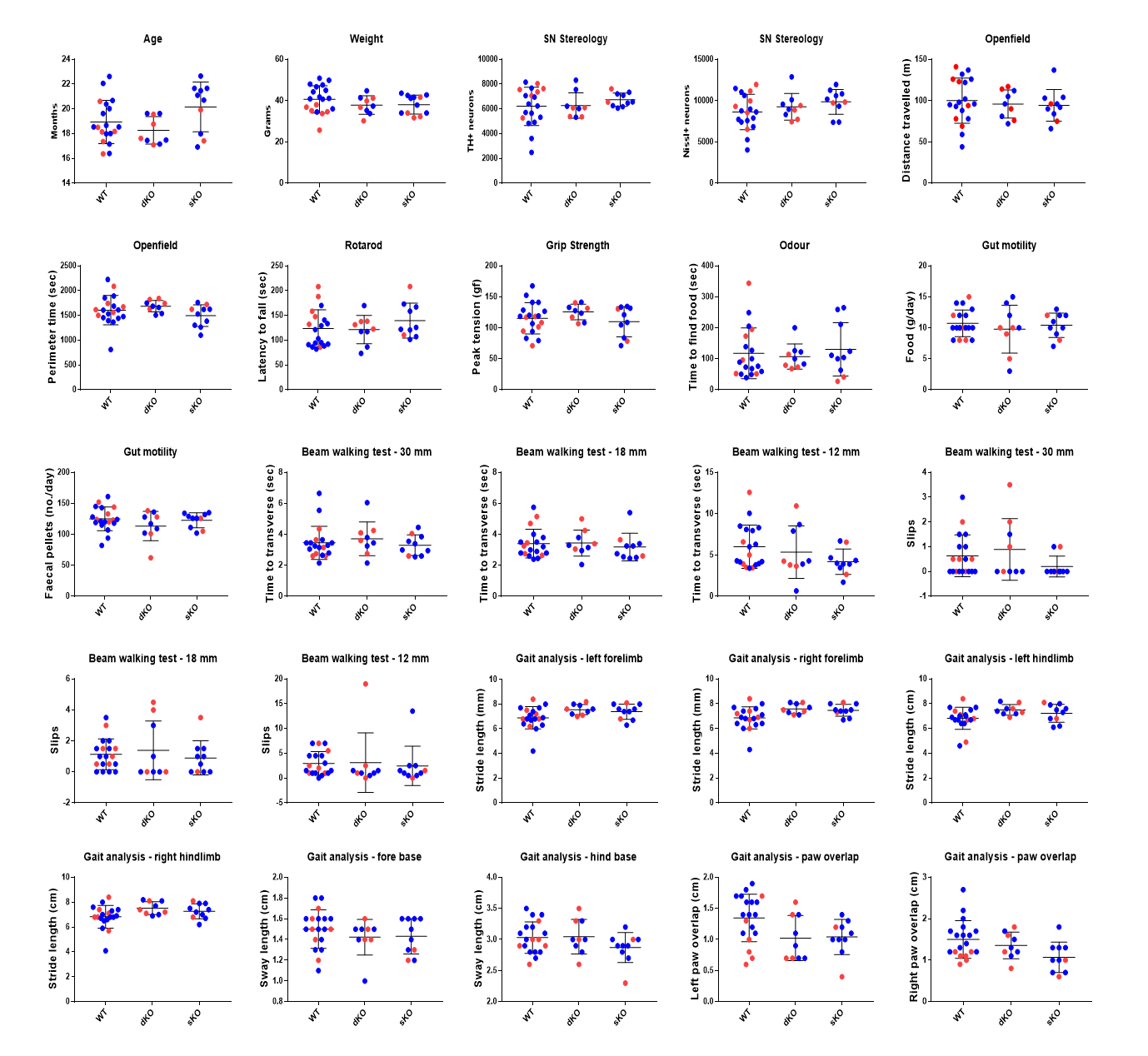


Supplementary Figure 11. Dopaminergic neurons in the substantia nigra of single *Pacrg* knockout mice. Representative images of dopaminergic neurons of the substantia nigra of single *Pacrg* knockout (sKO) and wildtype (WT) animals. Genotype blind unbiased counts of tyrosine hydroxylase (TH+) stained neurons was performed. Scale bar 100 μm.


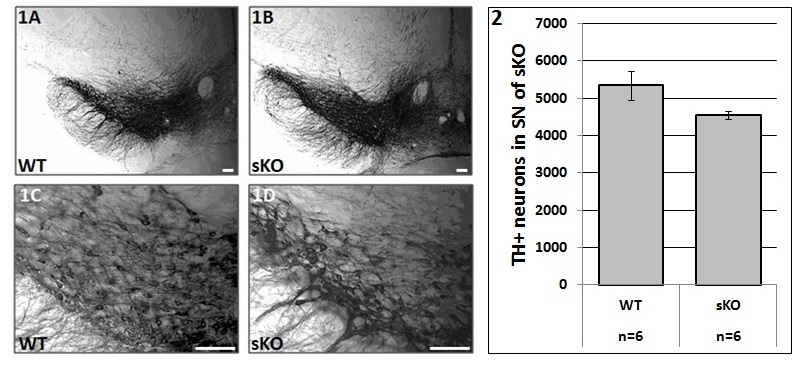


Supplementary Figure 12. Dopaminergic neurons in the substantia nigra of double *Park2*-*Pacrg* knockout mice. Representative images of dopaminergic neurons of the substantia nigra of double *parkin*-*Pacrg* knockout (dKO) and wildtype (WT) animals. Genotype blind unbiased counts of tyrosine hydroxylase (TH+) stained neurons was performed. Scale bar 100 μm.


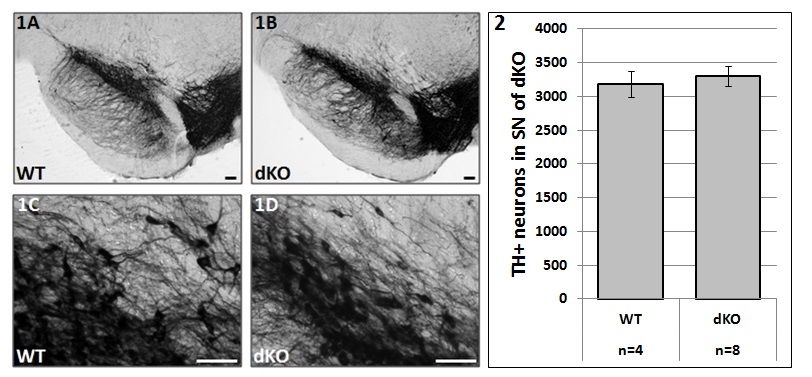

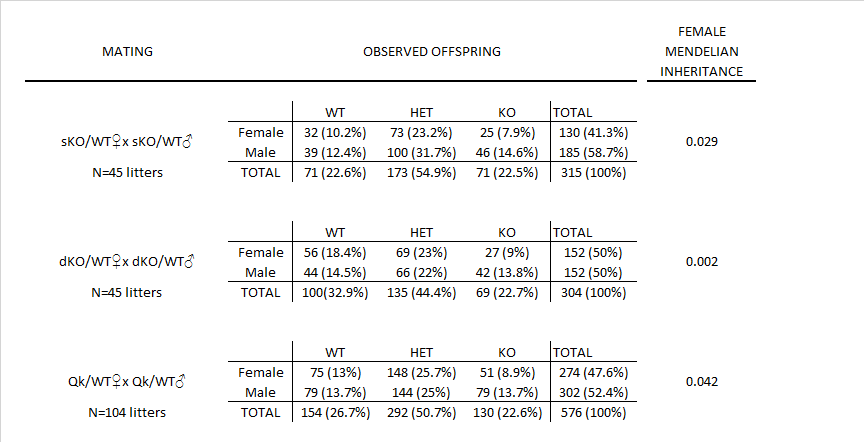


Supplementary Figure 13. Fewer female mice weaned harbouring two null *Pacrg* alleles.

Chi squared test suggested that the Mendelian ratios of females deviated from what was expected. Single *Pacrg* knockout (sKO), double *parkin*-*Pacrg* knockout (dKO) and wildtype (WT) animals, quaking viable (Qk) - a naturally occurring deletion of parkin and pacrg with dysregulated quaking expression).
